# Phenylketonuria - genetic, clinical and therapeutic aspects

**DOI:** 10.1186/1753-6561-6-S4-P6

**Published:** 2012-07-09

**Authors:** A Murariu, R Magopet, SO Salceanu, O Murariu, E Petrescu

**Affiliations:** 1 University of Medicine and Pharmacy ”Grigore T. Popa”, Iasi, Romania; 2 “Saint Spiridon” University Laboratory, Iasi, Romania

## Introduction

Phenylketonuria (PKU) is an autosomal recessive inborn error of phenylalanine (Phe) metabolism caused by mutations in phenylalanine hydroxylase (*PAH*) gene. It is one of the most prevalent metabolic disorders, with a high frequency (around 1/10,000) among Caucasians.

## Biochemistry and genetics of PKU

The normal route for Phe metabolism is its hydroxylation to tyrosine, catalyzed by PAH and requiring tetrahydrobiopterin as a cofactor. The human *PAH* gene is located on chromosome 12q23.2 and contains 13 exons. More than 500 disease-causing mutations have been identified in patients with PKU or hyperphenylalaninaemia (HPA), 67% of them being missense mutations. Most of them result in severe enzyme deficiency generating the PKU phenotype, but some are associated with a ”non-PKU HPA” phenotype. Since the *PAH* gene is biallelic, most patients are compound heterozygotes.

## Clinical manifestations and pathogenesis of PKU

Untreated PKU is associated with an abnormal phenotype including growth failure, microcephaly, seizures and intellectual impairment. The pathogenesis of these abnormalities involves the neurotoxic effects of hyperphenylalaninaemia, which are linked to Tyr deficiency (leading to a decrease in catecholamine neurotransmitters), the effect of elevated Phe concentrations on transport of other aminoacids across the blood brain barrier, accumulation of toxic by-products of Phe metabolism. Magnetic resonance imaging (MRI) has revealed white matter lesions in the brain of adult PKU patients, including hypomyelination and demyelination.

## Diagnosis of PKU

Newborn screening programs exist in many countries, based on the detection of a high blood Phe concentration. Conventional PKU diagnosis is based on the aberrant metabolic phenotype and the detection of disease causing mutations at the *PAH* locus. Available methods include Southern blotting, restriction enzyme digestion, gene sequencing. PKU mutation analysis is particularly useful in the detection of carriers, for prenatal diagnosis.

## Treatment of PKU

The foundation of PKU treatment is a low Phe diet which prevents the development of the neurological and psychological changes. It is recommended that dietary restriction should be started early (within one month of birth) and be followed for life. The existence of newborn screening programs and early dietary intervention allow children with PKU to live relatively normal lives.

